# Implementation of an on-site simulation programme during COVID-19 and the assessment of its impact on medical students’ competence

**DOI:** 10.1007/s11845-022-03057-z

**Published:** 2022-06-22

**Authors:** Niall James McInerney, Mohammad Faraz Khan, Laoise Coady, Jeffrey Dalli, Maurice Stokes, Suzzane Donnelly, Helen Heneghan, Ronan Cahill

**Affiliations:** 1 UCD Centre for Precision Surgery, Mater Misericordiae University Hospital, Eccles St, Dublin 7, Ireland; 2 UCD School of Medicine, Belfield, Dublin 4, Ireland; 3 St Vincent’s University Hospital, Elm Park, Dublin 4, Ireland

**Keywords:** Competence, Medical education, Simulation

## Abstract

**Background:**

COVID-19 has greatly impacted medical students’ clinical education. This study evaluates the usefulness of a rapidly implemented on-site simulation programme deployed to supplement our disrupted curriculum.

**Methods:**

Students on surgical rotations received 4-hour tutor-led simulated patient sessions (involving mannikins with remote audio-visual observation) respecting hospital and public health protocols. Attitudes were questionnaire-assessed before and after. Independent, blinded, nonacademic clinicians scored students’ clinical competencies by observing real patient interactions using the surgical ward assessment tool in a representative sample versus those completing same duration medicine clinical rotations without simulation (Mann–Whitney *U* testing, *p* < 0.05 denoting significance) with all students receiving the same surgical e-learning resources and didactic teaching.

**Results:**

A total of 220 students underwent simulation training, comprising 96 hours of scheduled direct teaching. Prior to commencement, 15 students (7% of 191 completing the survey) admitted anxiety, mainly due to clinical inexperience, with only two (1%) anxious re on-site spreading/contracting of COVID-19. A total of 66 students (30%, 38 females and 29 graduate entrants) underwent formal competency assessment by clinicians from ten specialties at two clinical sites. Those who received simulation training (*n* = 35) were judged significantly better at history taking (*p* = 0.004) and test ordering (*p* = 0.01) but not clinical examination, patient drug chart assessment, or differential diagnosis formulation. Of 75 students providing subsequent feedback, 88% stated simulation beneficial (notably for history taking and physical examination skills in 63%) with 83% advocating for more.

**Conclusion:**

Our rapidly implemented simulation programme for undergraduate medical students helped mitigate pandemic restrictions, enabling improved competence despite necessarily reduced clinical activity encouraging further development.

## Introduction

The provision of medical education while not universally standardised has maintained a core structure by tradition for many years, primarily based on Flexnerian principles [1–3]. Students spend their initial years learning from a predominantly didactic curriculum. They then apply their theoretical knowledge in a clinical setting while also learning practical skills [4] in their final years. This structure has been employed successfully in our university and clinical partner sites for a long period of time. Due to the COVID-19 pandemic and a national public health directive, all on-site clinical medical education at our healthcare institutions ceased in March 2020, greatly disrupting student education. All face-to-face student-patient contact was halted to mitigate the risk of infection spread and instead students participated in online and self-directed learning, as at that time, those on-site had completed sufficient clinical experiences to progress. While some on-site attendance was re-permitted by the time of our next student rotations in August 2020, hospital and public restrictions continued to greatly restrict clinical access for students. As experiential learning is a fundamental component of educating competent physicians [5–7], we moved to implement an on-site simulation programme to help the next tranche of clinical students who had missed out on all clinical learning opportunities due to the preceding ban.

Crucial to any educational innovation, most especially those occurring near qualification, is the assurance of benefit. Competence (defined as “the quality or state of having sufficient knowledge, judgment, or skill” [8]) is a key requirement of new doctors as assessing the acquisition of knowledge alone ignores the varied skills required by clinicians [9]. The goal of competency-based medical education is to produce “health professionals who practice at a defined level of proficiency, in accord with local conditions to meet local needs” [10]. After gaining key competencies, students can, of course, progress onto proficiency and ultimately even excel in their chosen field but the key qualification standard is competence.

Simple establishment of a simulation programme alone, of course, is not a guarantee of useful education or new programme efficacy. Nor indeed is self-reported student satisfaction or confidence. While important at any time, maintenance of medical student standards is especially critical at a time when such students face commencing work during a healthcare crisis. Therefore, alongside implementation, here we detail the independently-assessed competency assessment of our rapidly established simulation programme. Importantly, the competency assessment was performed on actual hospital in-patients. While student curricula need to be equitable for all, the disruption of the pandemic and the structure of our curriculum in terms of summative assessment provided a unique opportunity to implement and assess the new educational component while also allowing all students to complete the simulation programme prior sitting their formal in-module university examinations.

## Methods

### Study participants

The focus of this study was to assess the attitudes and usefulness of a new simulation programme for senior undergraduate students commencing their clinical learning in general medicine and surgery following 2 or 3 years of preclinical education for graduate entry and direct undergraduate medical students, respectively. Students at this stage are divided into groups balanced for gender, nationality, and educational experience (graduate entry vs. undergraduate entry) and assigned to clinical attachments in surgery or medicine in equal proportions for 6 weeks, with groups then swapping over for the next 6 weeks. The cohort of students who started the academic year on the surgery rotation was the intervention group, and students who commenced on medicine were the control group. No academic parameters such as grade point average or prior examination results are taken into account for group assignment. Surgical attachments are each 1 week long, with all students rotating through a different surgical specialty each week in groups of five or six, with those in medicine also being attached to a clinical team each day. All students share a common lecture series in both medicine and surgery, and all have completed general clinical skills training, including history taking and examination, prior to commencement of such attachments. Overall the surgery and medicine modules together account for 40 credits that contribute to the students graduating grade point average (GPA), and the attachments in both medicine and surgery take place in five clinical locations, including two major university teaching hospitals (which host approximately half the class at any one time). Due to COVID-19 restrictions, this year, clinical accessibility was greatly reduced with only one student being allowed on each ward round. The outpatient experience was also greatly curtailed over the duration of the study, with most patients being seen virtually rather than in-person without student attendance.

This research has been approved for human subjects with low risk ethical exemption. This is anonymous data, with students recruited from one school. Permission has been obtained from the head of the school (UCD Surgery) to collect this data.

### Simulation facility and programme

The university’s section of surgery and surgical specialties has access to decommissioned operating theatres within one of our clinical sites. With the support of the hospital board and university section leadership, a simulation space was enabled, including the installation of some dedicated equipment. Basic mannikins were used as simulated patients with appropriate vital signs and medications listed in end-of-bed notes. Adjuncts (urinary catheters, nasogastric tubes, and venous-thromboembolic disease prophylaxis) were available to the students as required. A Smots™ (Scotia Medical Observation and Training System, Scotia UK plc, Edinburgh, UK) system was sourced to provide a remote observation audio-visual system that allowed communication with students by a simulation facilitator from an adjacent room.

The simulation programme focused on surgical history taking and synthesis as well as care plan formulation for an acutely presented patient in five clinical scenarios (see Table 1) as well as elective ward rounding. Students were allotted a 4-h surgical tutor-led simulated patient session, run biweekly in small groups respecting hospital and public health protocols. In sessions, students within each group were randomly paired and worked collaboratively to perform a history and examination, formulate a differential diagnosis, order appropriate laboratory and radiological investigations, and resuscitate/stabilise the patient. Following this, they composed a short and intermediate-term management plan. These scenarios incorporated skills required for both acute presentations and routine ward rounds. When all students completed the simulation, the simulation facilitator debriefed the students on the positive and negative aspects of their performance.

**Table 1:** Clinical scenarios used during simulation teaching sessions

**Scenario number**	**Scenario description**
1	Acute cholecystitis
2	Acute appendicitis in a 30-year-old female
3	Perforated diverticulitis with peritonitis
4	Acute pancreatitis
5	Small bowel obstruction secondary to adhesions

### Study design

To assess the usefulness of these simulation sessions, we both assessed student attitudes by questionnaire to both clinical placements at this time (at term start) as well as to simulation (at term-end) and designed a prospective study regarding competency assessments of students who had and who had not undergone simulation teaching.

#### Attitude questionnaire

All students were invited to complete an anonymous questionnaire surveying their attitudes to clinical placements as well as to simulation after their simulation sessions on a voluntary basis. The first surveyed their self-perceived anxiety and preparedness for assessing patients both by history taking and physical examination, and the second, whether they found simulation training beneficial. Students were informed that feedback provided would guide future iterations of this curriculum and also be used to enhance their own learning opportunities over the remainder of their undergraduate education.

#### Competency assessment

**Fig. 1:**
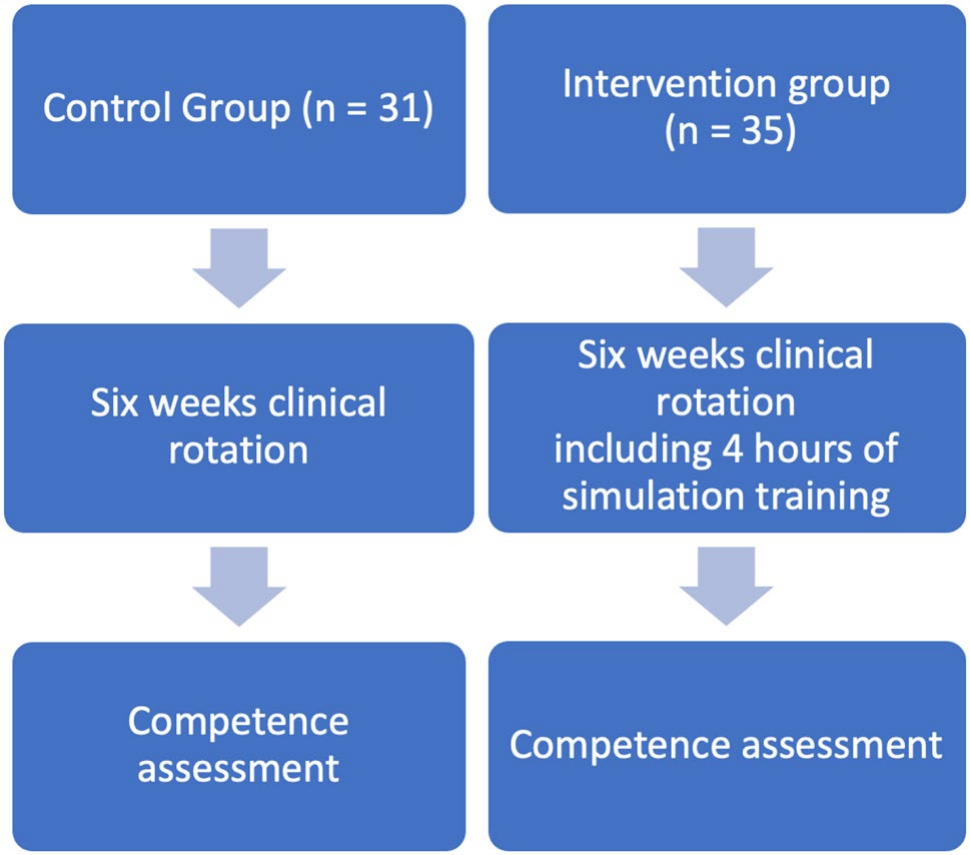
Study design (attached as Supplementary file)

Representative groups who completed simulation were compared against students who partook in a similar duration medical rotation without simulation (see Fig. 1) over the course of 1 week across the two university teaching hospitals after completion of the simulation sessions for the first 120 students. Students who were rotating through our other clinical sites at the time of assessment (approximately 50% of the class) were not included. Both groups of students had been attached to clinical teams for 6 weeks on a restricted basis as above and all shared common medicine and surgery e-learning and web-based resources but only the intervention group received simulation-based teaching. For the assessment, students were directly observed assessing real consenting patients with the objective of performing a global assessment and management plan formulation. Objective competency assessment was performed by nonacademic clinical staff. The twelve component SWAT [11] (Table 2) was used by the clinical observers who were blinded to whether the students had undergone simulation training or not. Student participants were informed that they were taking part in a formative assessment that would have no impact on the grade and was not informed of the components of the SWAT before the assessment.

**Table 2:** Surgical ward round assessment tool (SWAT) components and grading system

**Tasks**					
Takes history from the patient			Reviews drug chart		
Examines the patient			Checks nutrition status		
Checks patient is wearing appropriate thromboembolic deterrent stockings			Discusses differential diagnosis		
Checks vital signs and fluid charts			Checks wound (if applicable)		
Checks blood investigation results			Checks drain (if applicable)		
Checks imaging investigation results			Follows infection control protocols		

**Grading system**					
Well below expectations	Below expectations	Meets expectations		Above expectations	Well above expectations
1	2	3		4	5
Fails to complete task	Task completed with prompting	Task completed consistent with level		Task completed better then level	Task completed to junior doctor standard

Verbal patient consent was obtained for all assessments.

### Statistical analysis

The collected data was interrogated for normality (Shapiro–Wilk test *p* < 0.05) and confirmed to be nonparametric in nature. The difference between simulation and control groups was thus subsequently evaluated using the Mann–Whitney *U* test (*p* < 0.05), with each component of the SWAT being compared separately. Analysis was carried out using IBM SPSS, version 26 (NY, USA).

## Results

A total of 220 students underwent simulation training this semester, comprising over 96 hours of scheduled direct teaching. A total of 191 students (111 females and 90 graduate entries) were surveyed prior to commencing simulation training. A total of 22 students (10%) had prior experience in simulation training, mainly from student surgical and emergency medicine society extra-curricular events. A total of 15 students (7%) were anxious about upcoming ward-based learning, predominantly due to a lack of clinical experience. Only two students (1%) were anxious due to the risk of spreading/contracting COVID while on-site.

A total of 66 students (38 females and 29 graduate entries) were formally assessed by nonacademic clinical staff from ten specialties at two clinical sites. A total of 35 students had received simulation versus 31 who had not. Wound and drain site inspection and nutritional status were not applicable to all patients, and as such were removed from the final analysis. Competency assessment scores for each component of the SWAT can be seen in Table 3. Students who received simulation training (*n* = 35) were significantly better at history taking (*p* = 0.004) and appropriate laboratory (*p* = 0.001) and radiological investigation (*p* = 0.01) ordering. There was no significant difference between the groups otherwise, including clinical examination, assessing the patient’s drug chart, and differential diagnosis formulation.

**Table 3:** SWAT competence assessment scores and their statistical analysis of intervention (simulation) versus control (no simulation) student groups by blinded, independent clinicians

**Task**	Simulation group (*n* = 35)	Control group (*n* = 31)	*P*-value
Take a history from the patient	3.69 ± 0.76	3.19 ± 0.70	0.004
Examine the patient	3.37 ± 0.77	3.06 ± 0.85	0.120
Check patient is wearing appropriate thromboembolic deterrent stockings	2.86 ± 1.00	3.10 ± 1.30	0.557
Check vital signs and fluid charts	3.43 ± 0.88	3.23 ± 0.80	0.329
Check blood investigation results	3.51 ± 0.70	2.84 ± 0.78	< 0.001
Check imaging investigation results	3.11 ± 0.72	2.68 ± 0.70	0.010
Review drug chart	3.40 ± 0.91	3.00 ± 0.58	0.065
Discuss differential diagnosis	3.40 ± 0.91	3.16 ± 0.73	0.074
Follow infection control protocols	3.97 ± 0.82	3.39 ± 1.28	0.067

A total of 75 students (34% of the class; 22 males and 53 females, 37 undergraduate and 38 graduate entries) provided feedback at the conclusion of the semester. Overall, 88% of surveyed students found simulation training beneficial, with 82.7% advocating for more simulation training to be added to the curriculum. A total of 62.7% of surveyed students reported that simulation training had a positive impact on their history-taking ability, with 4% saying that simulation training had a negative impact on their history taking ability without explaining why. A total of 60% of surveyed students felt simulation training had a positive impact on their ability to perform a physical examination, with 9.3% saying simulation training had a negative impact. The majority of surveyed students felt more prepared assessing surgical patients, with 84% feeling more prepared seeing patients on ward rounds and 79% feeling more prepared in the outpatient clinic, however, only 48% felt more prepared in the emergency department.

## Discussion

Medical simulation allows students to interact with a “device that presents a simulated patient (or part of a patient) that interacts appropriately with the actions taken by the simulation participant.” Thus, the interaction mimics what occurs with actual patients but in a safe and controlled environment [12]. During the pandemic, simulation allows students to apply and practice their skills under close supervision without the same risks of infection to themselves and others as would occur in the hospital setting. Simulation programmes require careful planning on a number of fronts [13, 14]. Adequate facilities and equipment must be sourced. Clinical scenarios must be planned, education goals must be discussed, and most importantly, capable faculty must be available [15, 16]. While our institution had already commenced such planning as part of ongoing curriculum modernisation, the immediate requirement for supplementing education during the pandemic meant the implementation of our simulation programme had to be expedited.

This study shows that such rapid implementation of a simulation programme can augment an existing but disrupted curriculum. Despite our simulation programme being low fidelity (due to limited budget), it appears effective. While it can be difficult to define and objectively appraise clinical competence [17], nonacademic clinical staff provide an excellent objective assessment of the skills required to be a competent doctor. Inclusion of actual patients (all of whom were screened negative for COVID-19 as part of their admission protocol) in the observation study ensures a real-world test-of-usefulness for the competency assessment. The benefits of such a single simulation session were confined in our experience to history taking and radiological and laboratory investigation but not interestingly in other areas such as venous thromboembolism prophylaxis, which was a definite component of the simulation session. Generally, other than this, scores tended to be higher for each component in those receiving simulation versus those who did not suggest, perhaps a type 2 error is present and larger numbers could have detected a difference.

By simulating common surgical scenarios, the majority of students also felt more confident and capable when dealing with real-life patients on day one of their careers and recommended continuing the development of the programme. Interestingly, some students, however, reported negative effects of the initiative although the reasons are unclear, although we have previously found a minority of students opposed to other evolutions in the curriculum or assessment programmes previously. Interestingly, too, only two students (1%) were anxious about spreading and/or contracting COVID-19. While this may be a reflection of generational attitudes to the pandemic or alternatively student eagerness for a return to clinical education superseding their concerns, their increased awareness of infection prevention and control measures in our institutions may simply have the students feeling safeguarded against infection. Notably, of the twelve components of our competence assessment, students in both groups scored highest on infection prevention and control measures, reflecting their increased awareness and compliance.

Despite the increasing interest in simulation, including due to the pandemic, there is still rather limited evidence for the objective performance benefits of simulation training for medical students, most particularly in the surgical curriculum. While two other studies have shown that simulation training improves competence and performance when assessing simulated surgical patients [18, 19], our study shows that performance benefits gained from simulation training can be translated to interactions with real-life patients. The majority of studies done have instead focused on subjective self-perceived parameters such as confidence and enjoyment (20–22), which also seem to have generally be achieved by students in this study. While these are valuable metrics and important to our students, ultimately, medical students must become competent doctors. The transition from medical student to junior doctor can be daunting. Although they often have vast knowledge, this must be synthesised and applied to clinical scenarios while also acquiring and implementing newly acquired clinical skills. By improving medical students’ competence, this transition should be eased, enabling competent medical students to flourish and become proficient doctors.

A limitation of this study is the fact that the two groups differ with regard to clinical attachment. Exceptionally, the difference was minimised by the restrictions on clinical site education imposed by the pandemic, meaning that students on surgical rotations had less opportunity to gain surgical patient experience than usual. We were unable to randomise students as the university had previously divided students into groups, but as these groups are designed to incorporate a broad range of demographic features, this was felt to not impact our results. Also, there was a lack of a baseline assessment of each student prior to simulation training, which could have been used to gauge individual improvement following such training. Again, due to pandemic restrictions, we were unable to perform a baseline assessment prior to module commencement. However, this was our students’ first clinical attachment and so the baseline was likely to be similar, most especially with respect to clinical competence. Finally, not every student was assessed, again due to the constraints of the pandemic and the difficulties of moving students between clinical sites for non-essential purposes.

Therefore, in summary, this early investigative study has shown that simulation training can be effectively implemented into a disrupted curriculum, and its results will encourage and guide future iterations of our surgical curriculum as well as, hopefully, interesting others. While considerable tutor and surgical enthusiasm is required, it has proven to be a generally welcome addition to the curriculum by both staff and students. Most importantly, it has helped student performance at a very challenging time in their studies as well as for healthcare and medical education institutions in Ireland and globally.
